# Genetically Induced Cell Death in Bulge Stem Cells Reveals Their Redundancy for Hair and Epidermal Regeneration

**DOI:** 10.1002/stem.1910

**Published:** 2014-12-02

**Authors:** Iwona Driskell, Feride Oeztuerk-Winder, Peter Humphreys, Michaela Frye

**Affiliations:** Wellcome Trust—Medical Research Council Cambridge Stem Cell Institute, University of CambridgeCambridge, United Kingdom

**Keywords:** Adult stem cells, Epidermis, Stem cell plasticity, Targeted gene disruption

## Abstract

Adult mammalian epidermis contains multiple stem cell populations in which quiescent and more proliferative stem and progenitor populations coexist. However, the precise interrelation of these populations in homeostasis remains unclear. Here, we blocked the contribution of quiescent keratin 19 (K19)-expressing bulge stem cells to hair follicle formation through genetic ablation of the essential histone methyltransferase Setd8 that is required for the maintenance of adult skin. Deletion of Setd8 eliminated the contribution of bulge cells to hair follicle regeneration through inhibition of cell division and induction of cell death, but the growth and morphology of hair follicles were unaffected. Furthermore, ablation of Setd8 in the hair follicle bulge blocked the contribution of K19-postive stem cells to wounded epidermis, but the wound healing process was unaltered. Our data indicate that quiescent bulge stem cells are dispensable for hair follicle regeneration and epidermal injury in the short term and support the hypothesis that quiescent and cycling stem cell populations are equipotent. Stem Cells *2015;33:988–998*

## Introduction

Skin stem cells are established during development and retained in adulthood allowing the body to replace, restore, and regenerate lost, damaged, or diseased epidermal cells. To ensure the integrity of adult skin, epidermal stem and progenitor cells undergo hierarchical differentiation in which their lineage potential becomes increasingly restricted. However, a multipotent stem cell at the apex of epidermal cell hierarchy has yet to be identified.

The skin epidermis comprises the interfollicular epidermis, sebaceous glands, and hair follicles and these compartments are maintained by different populations of stem cell pools [1]. Whether a single multipotent epidermal stem cell population can in principle maintain these autonomous compartments in homeostasis is unknown, but all stem cell populations can be mobilized for tissue repair following injury [2]. The interfollicular epidermis is maintained by a combination of slow-cycling stem and committed progenitor cells [3,4]. In the hair follicle, quiescent stem cells reside in the bulge region (BG) and cycling stem cells in the hair germ beneath the bulge. Both types of stem cells are responsible for the cyclic regeneration of the hair follicle during homeostasis [5–7]. The sebaceous glands and the infundibulum, the part of the hair follicle that connects it to the interfollicular epidermis, are maintained by cycling stem cells [8–13].

The interrelationship between the stem cell pools of the distinct compartments remains unclear, yet one commonality is emerging: in the epidermis, stem cell populations can be either quiescent or active (more proliferative). It has been suggested that the more rapidly cycling progenitors may have a primary role in skin homeostasis, whereas the slow-cycling stem cells promote tissue repair [4,14]. The relationship between quiescent and more proliferative stem and progenitor populations is mostly studied in the hair follicle, as each hair follicle undergoes cycles of growth (anagen), apoptosis-driven destruction (catagen), and a resting period (telogen) [15]. The sequence of events that takes place in response to initiation of hair follicle growth is now well understood and begins with proliferation of cells located in the hair germ [16–19]. This intense phase of cell proliferation leads to an expansion of the lower portion of the hair follicle, followed by the differentiation into hair shaft and its inner root sheath [20,21]. Hair follicle bulge cells enter the cell cycle later and form the outer root sheath [5,22]. Although both populations act interdependent to regenerate the hair follicle, the hair follicle stem cells are dispensable for hair regeneration following laser ablation [5,23]. Whether quiescent and more proliferative stem and progenitor populations underlie a hierarchal structure in homeostasis remains unclear.

Here, we analyzed the morphological consequences of genetically induced cell death in quiescent K19-expressing bulge stem cells [24–26]. The advantage of our approach is that single bulge cells are depleted when recruited into the cell cycle, yet neighboring cells and the niche environment are left intact. To compromise the survival of stem cells, we conditionally deleted the histone methyltransferase Setd8/PR-Set7/KMT5a in K19-positive bulge stem cells. Setd8 is essential for chromosome stability during mitosis and its depletion causes cell cycle arrest and apoptosis in vitro and in vivo [27–34]. In adult skin, Setd8 is a crucial inhibitor of apoptosis and its activity is essential for cell survival and proliferation of long-lived epidermal progenitor cells, and its deletion causes the irreversible loss of sebaceous glands and interfollicular epidermis [34]. In contrast we now describe that induction of cell death in K19-positive bulge stem cells does not affect skin morphology. We conclude that quiescent bulge stem cells are dispensable for the formation of hair follicles and are not required for epidermal regeneration after injury.

## Materials and Methods

### Ethics Statement

All mouse husbandry and experiments were carried out according to the local ethics committee under the terms of a U.K. Home Office license (PPL80/2619 and PPL80/2231).

### Transgenic Mouse Lines and Treatments

To conditionally delete Setd8 in skin, mice containing floxed alleles of the *SETD8* gene (kindly provided by Danny Reinberg) were crossed with K19CreER [35] or K14-Cre mice (The Jackson Laboratory, Bar Harbor, Maine (http://www.jax.org/index.html)). In K14-Cre mice, Cre-recombinase is expressed under the control of the keratin 14 promoter leading to deletion of Setd8 in all basal, undifferentiated cells of the epidermis. In K19CreER mice, Cre-recombinase is fused to a mutated estrogen receptor domain and can be activated by application of 4-OHT leading to specific deletion of Setd8 in the hair follicle bulge [26]. To generate GFP-reporter lines to measure Cre-recombinase activity, the respective lines were crossed with CAG-CAT-EGFP mice, expressing enhanced GFP (EGFP) upon Cre-mediated recombination [36]. The mouse lines were genotyped as described previously [34]. To delete p53, the mouse lines were crossed to p53 null mice [50].

To activate K19CreER, 3–5-week-old mice were treated topically with 1.4 mg 4-OHT dissolved in acetone or acetone alone as a control every other day. For TPA treatment, 1 µg of TPA in acetone was applied topically to back skin on alternative days to 4-OHT. To measure proliferation, mice were injected with a dosage of 250 µg 5-ethynyl-2′-deoxyuridine (EdU; 2.5 mg/ml in phosphate buffered saline (PBS)) intraperitoneally. DNA LRCs were generated by repeated BrdU injections of neonatal mice at P10 and animals were chased as indicated [38]. Wound biopsies were carried out with a circular biopsy punch (5 mm or 3 mm) on the dorsal skin.

### Mouse Keratinocyte Culture and Time Lapse Analyses

Epidermal cells were isolated from mouse back skin and cultured as described previously [51]. Tat-Cre was applied to cells at a concentration of 4 µM for 8 hours. Time lapse imaging was performed using a Leica DMI6000 microscope. GFP fluorescence and transmitted light images were acquired using a ×20 objective at 30 minutes intervals. Phase and GFP images were also collected every 2 hours using an Incucyte Zoom, four positions per well. Confluence metrics were generated for GFP with an adaptive threshold of 3.5 (calibrated units).

### RNA Extraction and QPCR

RNA was extracted from the cultured epidermal cells using Trizol Reagent (Life Technologies (https://www.lifetechnologies.com/uk/en/home.html)) according to the manufacturers' instructions. Following RNA extraction, cDNA was made using SuperScript III Reverse Transcriptase (Life Technologies (https://www.lifetechnologies.com/uk/en/home.html)). RT-PCR was run using the standard protocol for TaqMan Fast Universal PCR Master Mix (2×) or Fast SYBR Green Master Mix using StepOne Plus Real-Time PCR System (Life Technologies (https://www.lifetechnologies.com/uk/en/home.html)). The standard amplification protocol was used with predesigned probe sets and TaqMan Fast Universal PCR Master Mix (2×; Life technologies (https://www.lifetechnologies.com/uk/en/home.html)). Primers used for SYBR Green QPCR were as follows: GFP forward (AGC AAG GGC GAG GAG CTG TT) and GFP reverse (GTA GGT CAG GGT GGT CAC GA), Setd8 forward (GTG TGA TCG CTA CCA AGC AGT TCT) and Setd8 reverse (ATA GTA CAT GTA GCA GCC AGT GGA GG), and GAPDH forward (GTC TCC TGC GAC TTC AAC AGC) and GAPDH reverse (TCA TTG TCA TAC CAG GAA ATG AGC). Expression of p53 was measured using the Taqman probe Mm01731287_m1. RNA levels were determined using the ΔCT method and relative expression levels were normalized to GAPDH.

### Tissue Staining and Antibodies

Tissue samples were either fixed overnight in 4% paraformaldehyde (PFA) and then embedded in paraffin or frozen unfixed, in OCT compound (VWR International (http://www.vwr.com)). Tail whole mounts were prepared following as previously described [38]. Paraffin (6–10 µm) and cryosections (10–100 µm) of back skin were used for immunostainings. After citrate epitope retrieval of paraffin sections, tissues were permeabilized for 5 minutes with 0.2% Triton X-100 at room temperature, blocked for 1 hour with 5% fetal calf serum (FCS), and incubated overnight with the appropriate antibody dilution. Stainings of cryosections were performed as for paraffin but after fixation for 10 minutes in 4% paraformaldehyde at room temperature. Tail epidermal whole mounts were prepared and immunolabeled as described previously [38]. To detect apoptotic cells in skin section, we used DeadEnd Fluorometric TUNEL System (Promega, http://www.promega.com) according to the manufactures instructions. To isolate bulge stem cells and their progenitors, flow cytometry for the cell surface markers CD34 and Itga6 was performed as described previously [52].

Primary antibodies against the following proteins were used: Itga6 (1:200; cd49f, AbD Serotec, http://www.abdserotec.com), Ki67 (1:100; SF6, Vector Laboratories, Ltd., https://www.vectorlabs.com/uk/default.aspx), cleaved Caspase-3 Asp175 (1:100; 9661, Cell Signaling, http://www.cellsignal.com), and Phospho-H2A. XSer139 (γH2AX) (1:400; 20E3 Cell Signaling, http://www.cellsignal.com), H4K20me1 (1:500; 39175/39180 Active Motif, http://www.activemotif.com/), BrdU (1:100; BU1/75 [ICR1], Abcam, http://www.abcam.com/), and GFP (1:500; ab13970, Abcam, http://www.abcam.com/). An Alexa594 conjugated antibody against keratin 15 (C8/144B) was a kind gift from Fiona Watt. Secondary antibodies were added at a dilution of 1:500 for 1 hour at room temperature together with DAPI to label nuclei. EdU was stained for using Click-iT EdU Alexa Fluor 647 Imaging Kit from Life Technologies (http://www.lifetechnologies.com) according to the manufactures instructions.

Brightfield images were acquired using an Olympus IX80 microscope and a DP50 camera. Confocal images were acquired on a Leica TCS SP5 confocal microscope. Z-stacks were acquired at 100–400 Hz with an optimal stack distance and 1,024 × 1,024 dpi resolutions. Z-stack projections were generated using the LAS AF software package (Leica Microsystems, http://www.leica-microsystems.com). Image analysis was preformed with ImageJ/Fiji software (http://www.micro-shop.zeiss.com). H&E and Ki67 images were collected using Zeiss Axioimager M2 with axiocam MRc camera and Axiovision software.

## Results

### Skin Morphology Is Unaffected by Ablation of Setd8 in Hair Follicle Stem Cells

**Figure 1:**
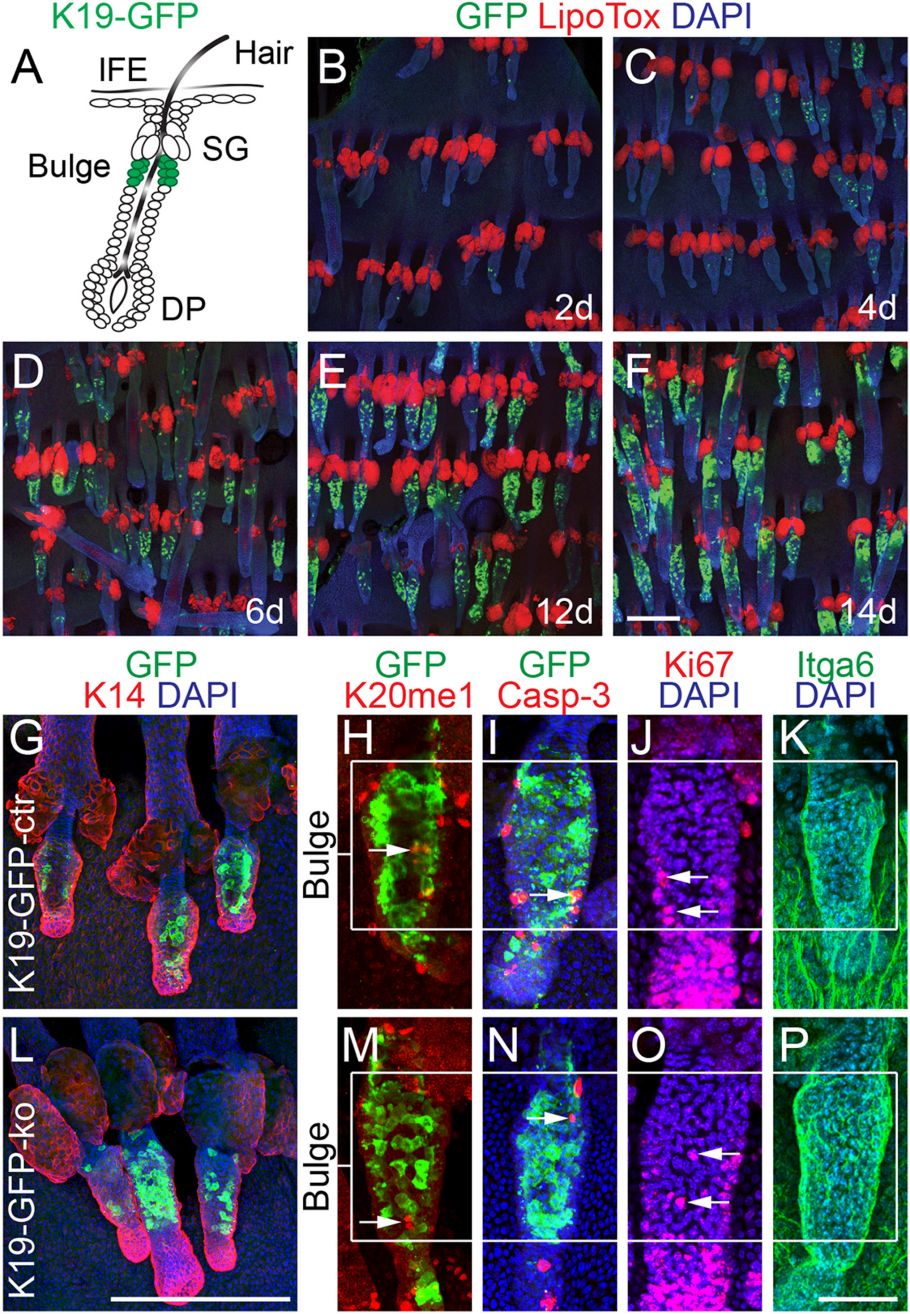
Hair follicle homeostasis is unaffected by deletion of Setd8. (A): Schematic diagram showing K19-Cre-induced deletion of Setd8 in the hair follicle bulge (green cells). (B–F): Time course of Cre-mediated recombination as measured by expression of the reporter GFP (green) using 4-hydroxy tamoxifen for 2–14 days (d) in tail skin whole mounts. Sebaceous glands are labeled with Lipo Tox (red) and nuclei are counterstained with DAPI (blue). Animals used for the experiment (B–F): Eight littermates at the age of 4 weeks. (G–P): Immunofluorescence labeling of keratin 14 (K14) (red) (G, L), H4K20 monomethylation (K20me1) (red) (H, M), cleaved caspase-3 (Casp-3) (red) (I, N), Ki67 (red) (J, O), and integrin alpha 6 (Itga6) (green) (K, P) in bulges of control (K19-GFP-ctr) (G–K) and Setd8-deleted (K19-GFP-ko) (L–P) hair follicles. Cre-recombination is visualized by GFP (green) (G, H, I, L, M, N) and nuclei are counterstained with DAPI (blue) (G, I, J, K, L, N, O, P). Animals used for the experiment (G–P): 18 age-matched mice at the age of 4 weeks (7 K19-GFP-ko; 11 K19-GFP-ctr). Scale bars = 200 µm (B–G, L) and 50 µm (H–K; M–P). Abbreviations: DP, dermal papilla; GFP, green fluorescent protein; SG, sebaceous gland.

To determine whether the self-renewing property of multipotent hair follicle stem cells required expression of Setd8, we caused its deletion in bulge stem cells using inducible Cre-recombinase (Cre) under the control of the K19-promoter [35]. To monitor Cre-activity in the bulge we used an inducible green fluorescent protein (GFP)-reporter (Fig. 1A) [36]. After treatment with 4-hydroxy tamoxifen (4-OHT) for 14 days, the vast majority of bulge cells in the tail skin stained positive for GFP (Fig. 1B–1F). The number of GFP-positive cells was comparable in control (K19-GFP-ctr) and Setd8 knockout (K19-GFP-ko) hair follicles (Fig. 1G, 1L), and deletion of Setd8 in quiescent bulge stem cells did not cause any adverse phenotype of the telogen hair follicles (Fig. 1G–1P).

Setd8 is the sole enzyme required to catalyze the formation of monomethylated histone 4 at lysine 20 (H4K20me1) [37]. We confirmed that H4K20me1 (K20me1) was absent in GFP-positive bulge cells (Fig. 1H, 1M; arrows), and that the number of apoptotic (cleaved Casp-3) or dividing (Ki67) cells as well as expression of cell surface markers (Itga6) enriched in bulge stem cells was unaffected by deletion of Setd8 (Fig. 1I–1K, 1N–1P).

**Figure 2:**
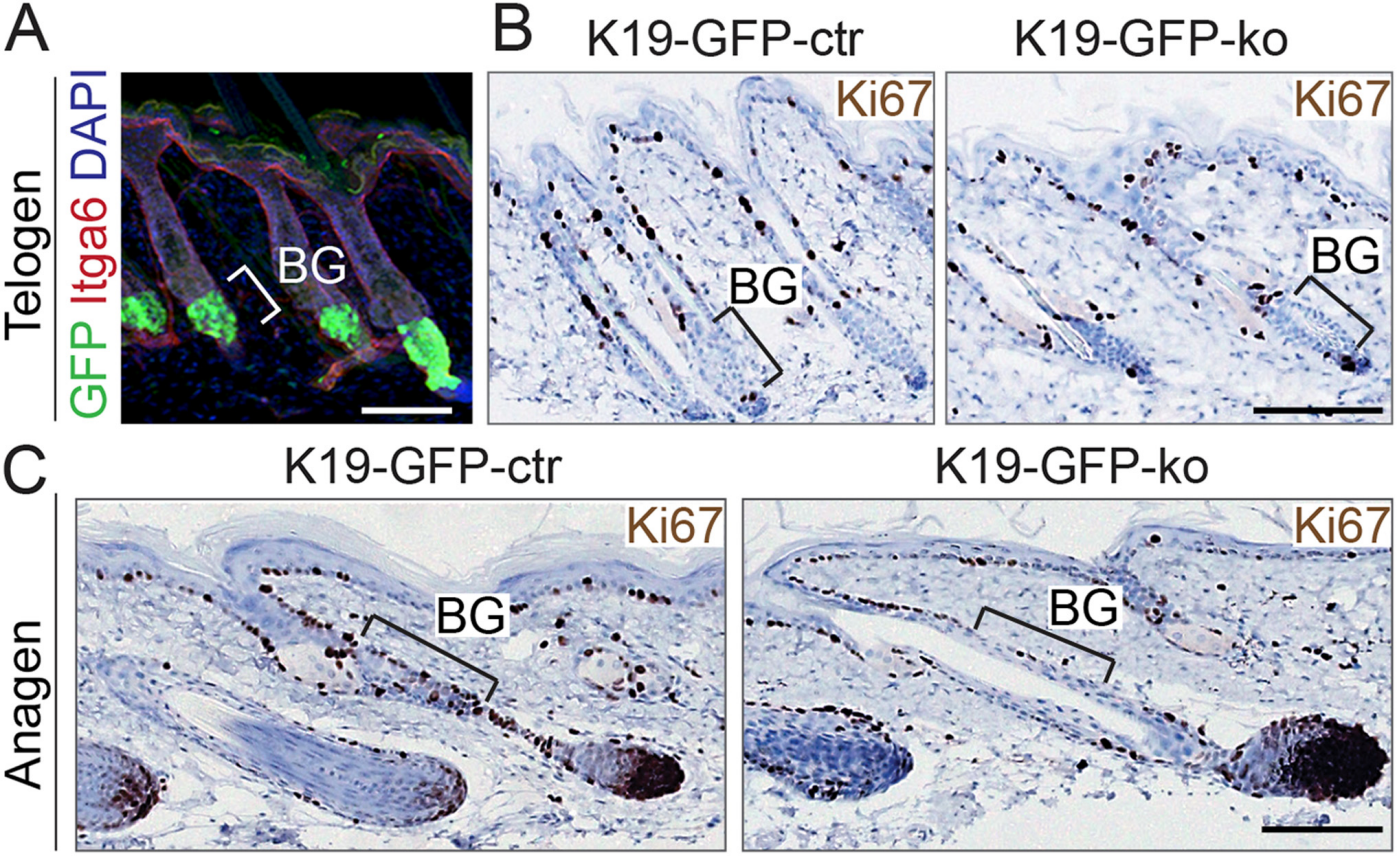
Number of proliferating cells in Setd8-deleted anagen bulges is reduced. (A): Effective Cre-recombination in back skin visualized by GFP (green). Sections are counterstained for Itga6 (red) and DAPI (blue). (B, C): Immunohistochemistry for Ki67 (brown) in telogen (B) and anagen (C) bulges (BG) using sections from control (K19-GFP-ctr) and Setd8-deleted (K19-GFP-ko) back skin. Animals used for the experiment: 18 age-matched mice at the age of 4 weeks (7 K19-GFP-ko; 11 K19-GFP-ctr). Scale bars = 100 µm (A–C). Abbreviations: BG, bulge region; GFP, green fluorescent protein.

Similarly to tail skin, we obtained a high degree of recombination in hair follicle bulges in the back skin (Fig. 2A), but did not observe any skin phenotype when Setd8 was deleted (Fig. 2B). Bulge stem cells only contribute to the generation of the hair follicle in its growing phase (anagen) [5], but also Setd8-deleted anagen hair follicles (K19-GFP-ko) did not exhibit any morphological differences (Fig. 2C). However, we observed a reduced number of proliferating Ki67-positive cells in the BG in the absence of Setd8 (Fig. 2C; line), indicating that Setd8 may be required for quiescent bulge stem cells to enter the cell cycle.

### Setd8-Depleted Bulge Cells Fail to Contribute to Hair Growth and Regeneration

**Figure 3:**
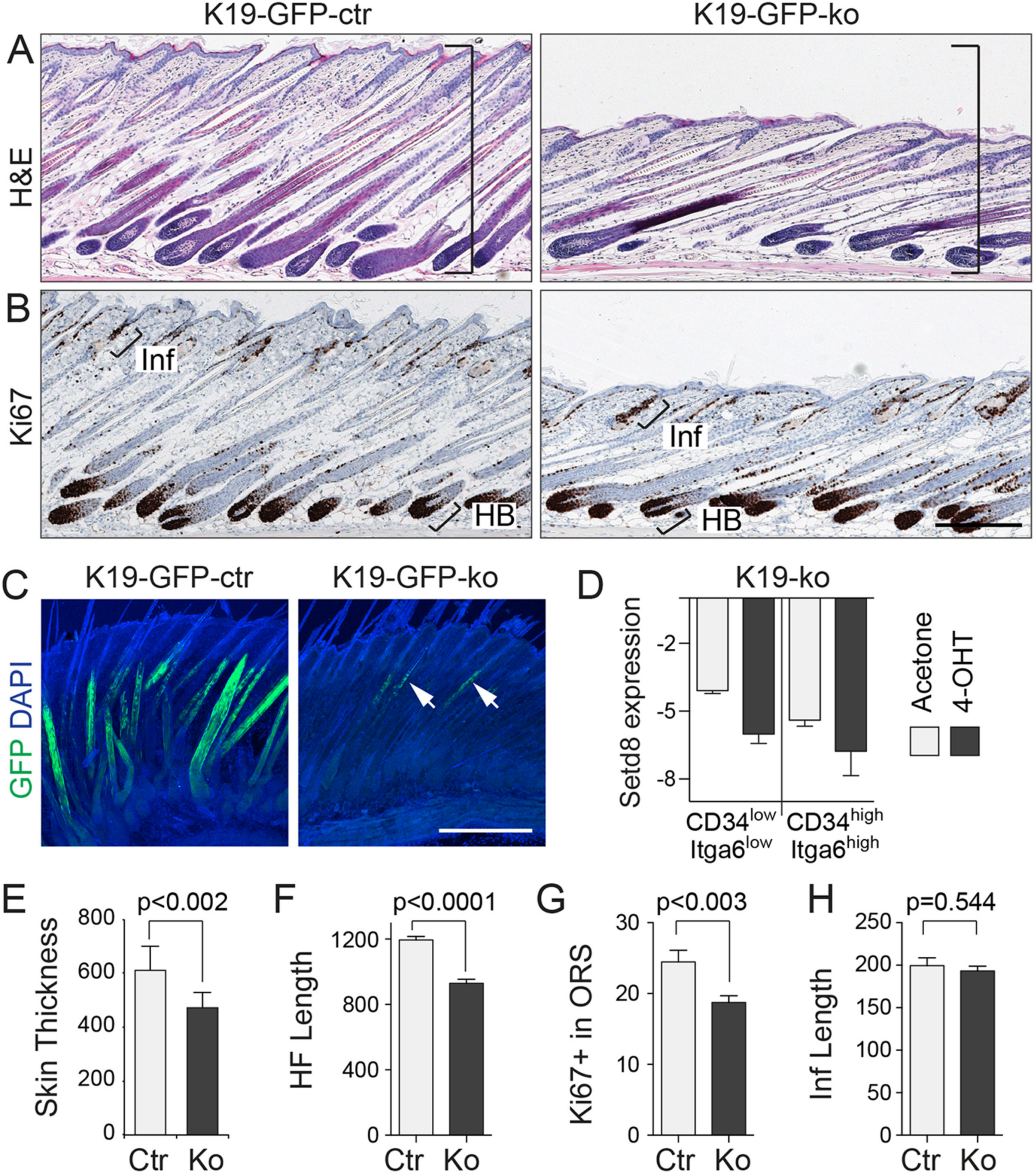
Hair follicle length is reduced when Setd8 is deleted in bulge stem cells. (A, B): Hematoxylin and Eosin (H&E) staining (A) and Ki67 (B) of back skin from control (K19-GFP-ctr; left panel) and Setd8 depleted (K19-GFP-ko; right panel) hair follicles following TPA/4OHT treatment. Bars in (A) indicate change in skin thickness between samples. (C): Immunofluorescence staining for GFP (K19 progeny) in back skin whole mounts of control (K19-GFP-ctr; left panel) and Setd8 depleted (K19-GFP-ko; right panel) sample following TPA/4OHT treatment. Arrows indicate GFP-positive progeny (C). Images in (A)–(C) were taken 9 days after the last treatment with TPA. (D): QPCR of Setd8 mRNA in bulge stem cells (CD34^high^/Itga6^high^) and their progeny (CD34^low^/Itga6^low^) isolated by flow cytometry from Setd8 knockout mice (K19-ko) treated with 4-OHT or acetone as a control. Graph represents ΔCT of GAPDH versus Setd8. Bars show mean with range estimated using pooled data from three technical and three (4-OHT) or two (acetone) biological replicates. (E–H): Quantification of skin thickness (E), hair follicle (HF) length (F), number of Ki67-positive cells in the outer root sheath (ORS) (excluding bulb) (G), and length of the infundibulum (Inf) (H). Error bars (E–H): SEM. *p*-Values were estimated using unpaired *t* test. Animals used for the experiment: Six age- and sex-matched mice (three ctr; three ko) at the age of 4 weeks treated for 14 days with 4-OHT and TPA (A–C; E–H). Five littermates (ko) at the age of 4 weeks treated for 14 days with 4-OHT or acetone and twice with TPA (D). Scale bars = 200 µm (A–C). Abbreviations: 4-OHT, 4-hydroxy tamoxifen; GFP, green fluorescent protein.

To recruit quiescent bulge stem cells into the cell cycle we exposed the back skin to the phorbol ester, TPA [38]. However, the combinatorial treatment of 4-OHT to delete Setd8 and TPA to induce anagen neither affected the entry into anagen nor caused major morphological differences in K19-GFP-ko mice (Fig. 3A). The overall epidermal cell proliferation in the hair bulb and the infundibulum (Inf) was similar in control and knockout mice (Fig. 3B). To analyze the fate of Setd8-depleted bulge stem cells, we examined the back skin for expression of the reporter GFP, and found that GFP-positive bulge cells contributed to the growing hair follicle in wild-type skin but failed to do so in the absence of Setd8 (Fig. 3C). The small number of GFP-positive Setd8 depleted cells located to upper part of the hair bulge (Fig. 3C, arrows). The nearly complete loss of GFP-positive Setd8-depleted bulge cells can be explained by cell death upon cell cycle entry [34] (Supporting Information Fig. S2D). We were unable to isolate GFP-positive Setd8-depleted bulge cells for any further analyses (not shown). To unequivocally demonstrate the effectiveness of our genetic modulation regime, we deleted Setd8 in the bulge by treatment with 4-OHT or acetone for 2 weeks. We then forced the cells into cell cycle by treating the back skin twice with TPA. We isolated bulge stem and progenitor cells by flow sorting for the cell surface markers CD34 and integrin alpha 6 (Itga6) [39,40]. In both stem cells (CD34^high^/Itga6^high^) and progenitors (CD34^low^/Itga6^low^), we measured reduced expression of Setd8 mRNA in 4-OHT treated hair follicles (Fig. 3D). Thus, our data indicated that bulge stem cells require Setd8 to contribute to hair growth. Moreover, our results also demonstrate that bulge cells can contribute to hair regeneration, yet they are not required for the formation of an anagen hair follicle.

While the overall skin morphology remained unchanged in the absence of Setd8 in the hair follicle bulge, we noticed a significant decrease in skin thickness when Setd8 was deleted in the hair follicle bulge (Fig. 3A; lines; Fig. 3E). In anagen hair follicles push themselves down into the dermis and the reduced skin thickness reflected a significant decrease in hair follicle length (Fig. 3F). The subtle phenotype of reduced hair follicle length might be explained by the lack of proliferating bulge cells and consequently the decreased number of proliferating Ki67-positive cells in the outer root sheath (Fig. 3G). As a control, we confirmed that the length of the infundibulum (Inf) was unaffected by deletion of Setd8 in the bulge (Fig. 3H).

**Figure 4:**
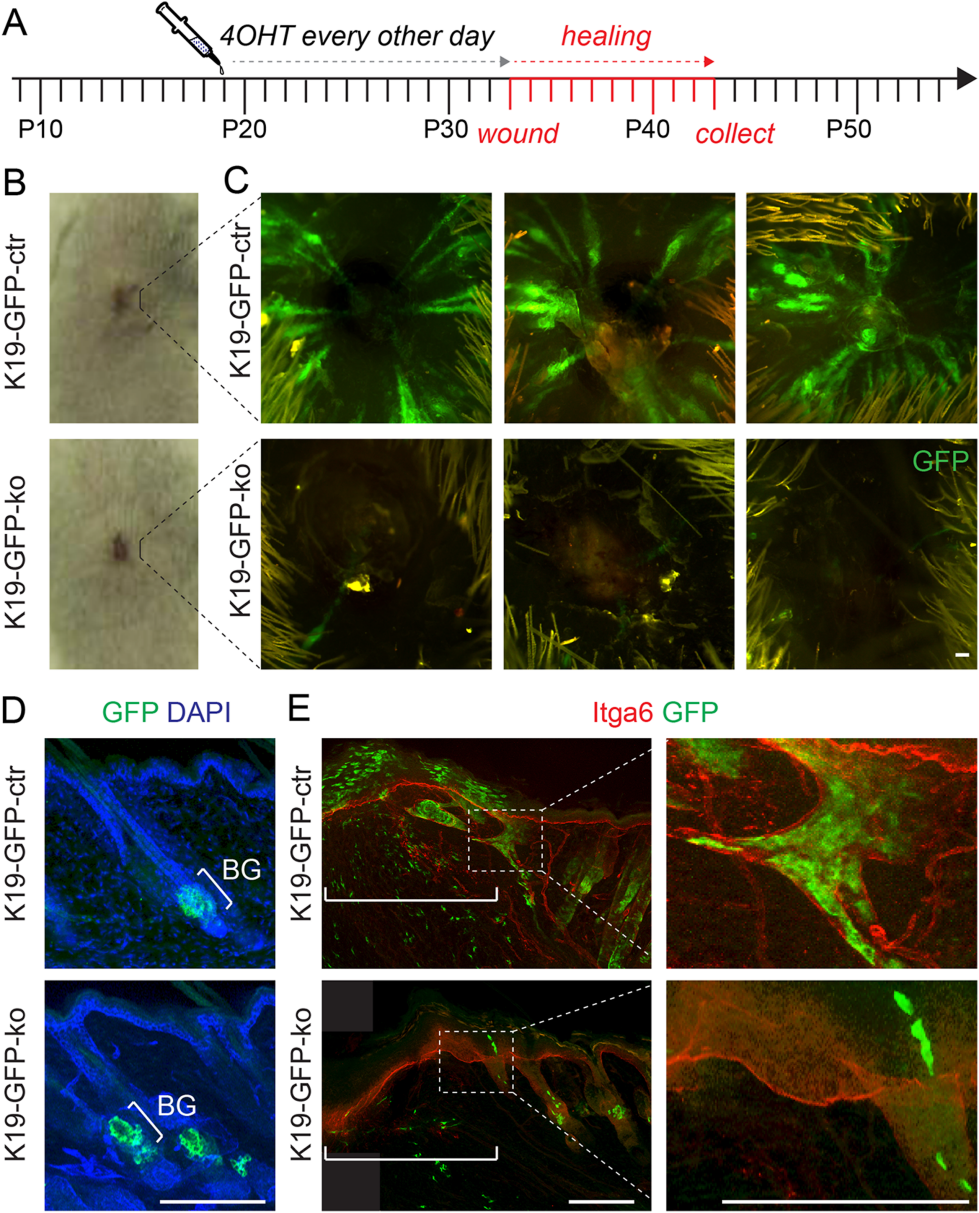
Wound healing is unaffected when Setd8 is depleted in the hair follicle bulge. (A): Schematic overview of the 4-OHT-treatment regime and wounding. (B): Wounded area in control (K19-GFP-ctr) (upper panel) and Setd8-knockout (K19-GFP-ko) (lower panel) mice. (C): Contribution of bulge cells (GFP; green) to wound closure in control (upper panel) and Setd8-knockout (lower panel) mice. Shown are three biological replicates. (D): Sections of back skin from control (upper panel) and Setd8-knockout (lower panel) animals with comparable GFP-labeling (green) in the bulge (BG). Cells are counterstained with DAPI (blue). (E): Immunofluorescence labeling for Itga6 (red) and GFP (green) of sections through the wound of control (upper panel) and Setd8-knockout (lower panel) skin. Lines demark the wound area and right hand panels show higher magnification of the boxed areas (dotted lines). Animals used for the experiment: eight age-matched mice at the age of 8 weeks (four K19-GFP-ko; four K19-GFP-ctr). Scale bars = 100 µm (C–E). Abbreviations: 4-OHT, 4-hydroxy tamoxifen; BG, bulge region; GFP, green fluorescent protein.

Our data are in line with the recent finding that hair follicle stem cells are dispensable for regenerating defective or damaged hair follicles [5]. However, bulge stem cells also contribute to the regeneration of the epidermis after injury [41], yet it is unknown whether they are required for wound healing. To test whether Setd8-depletion in bulge stem cells impaired epidermal regeneration, we performed wound-healing experiments (Fig. 4A). Wounds in K19-GFP-ko and K19-GFP-ctr mice healed with the same rates (Fig. 4B), yet only Setd8-expressing bulge cells were able to migrate into the wound (Fig. 4C). We confirmed that both knockout and control bulges exhibited the same level of recombination at the beginning of the experiment (Fig. 4D). Skin sections of the wounded areas further confirmed the lack of bulge-derived cells in K19-GFP-ko mice (Fig. 4E). While we cannot exclude that some bulge cells escaped recombination and contributed to epidermal wound healing, our data strongly indicate that bulge stem cells do not play a major role in epidermal regeneration after wounding.

### Deletion of p53 Fails to Rescue Setd8-Depleted Bulge Stem Cells

**Figure 5:**
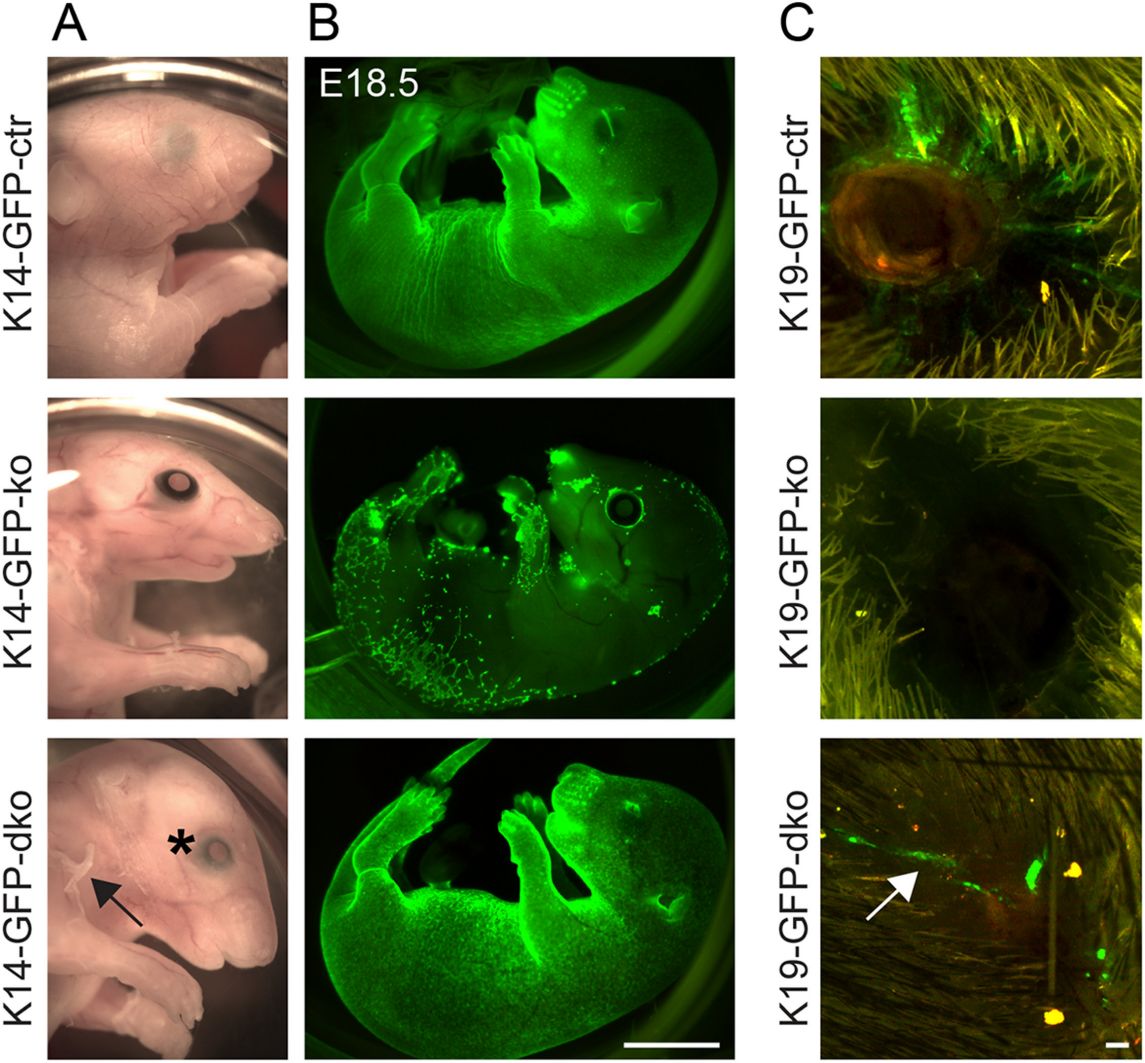
Simultaneous deletion of Setd8 and p53 only partially rescues cell death. (A, B): Photographic images (A) and reporter expression (GFP; green) (B) of E18.5 control (K14-GFP-ctr) (upper panels), epidermal deleted Setd8 (K14-GFP-ko) (middle panels), and p53 and Setd8 double deleted (K14-GFP-dko) (lower panels) animals. The arrow marks areas where skin peeled off. The asterisk marks partial eyelid closure. (C): Wound healing in control (K19-GFP-ctr) (upper panels), bulge Setd8-depleted (K19-GFP-ko) (middle panels), and p53 and Setd8 double deleted (K19-GFP-dko) (lower panels) animals. Arrow in lower panel indicates minor contribution of bulge cells (GFP; green) to wound healing. Animals used for the experiment: 13 age-matched mice at the age of ∼8 weeks (6 K19-GFP-ctr; 3 K19-GFP-ko; 4 K19-GFP-dko). Scale bars = 5 mm (B) and 100 µm (C). Abbreviation: GFP, green fluorescent protein.

**Figure 6:**
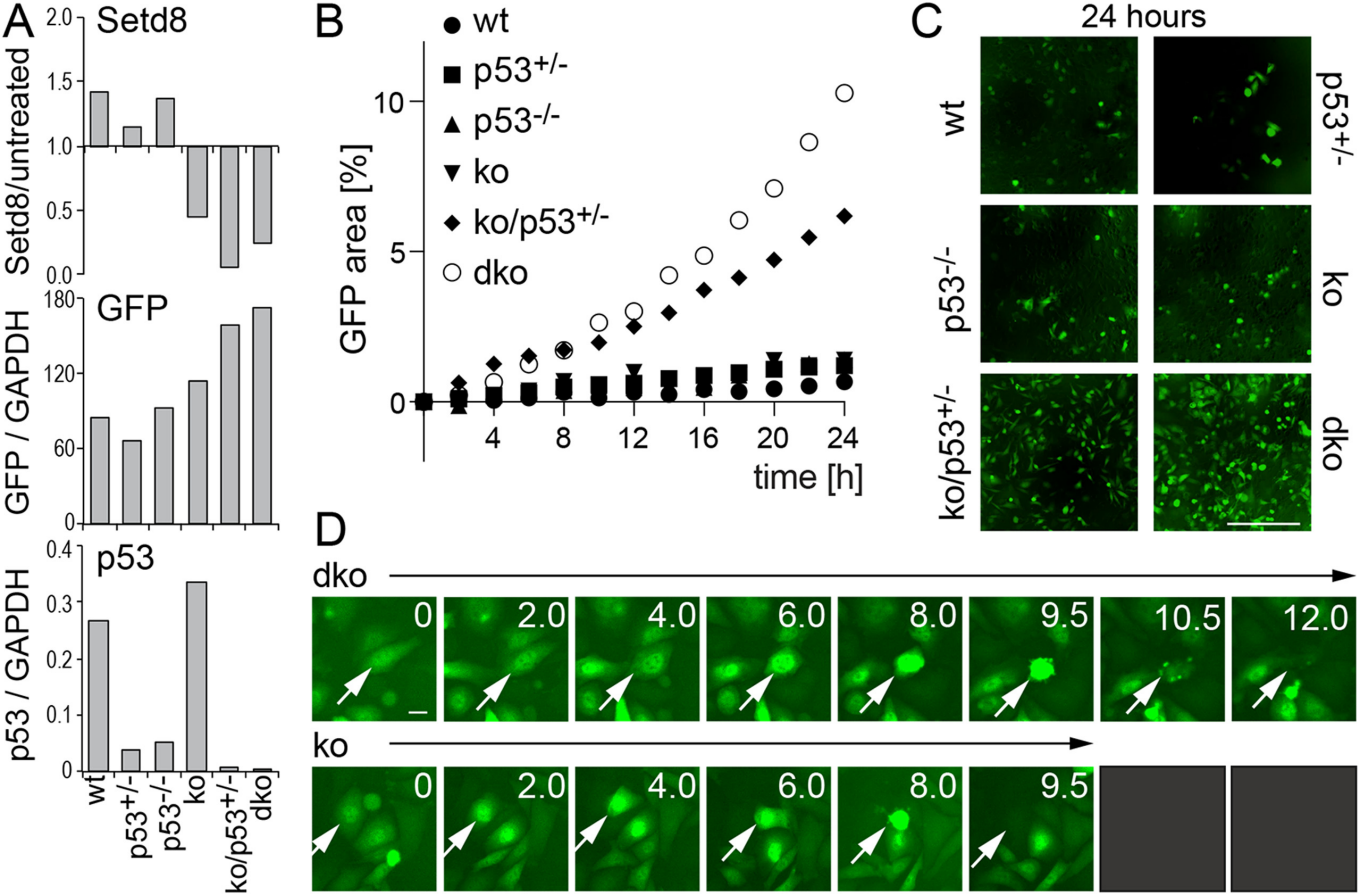
Simultaneous deletion of Setd8 and p53 does not rescue cell survival in the long term. (A): QPCR measuring Setd8, GFP, and p53 mRNA in the indicated cultured epidermal cells. Reduction of Setd8 expression is shown relative to untreated controls (upper panels). GFP and p53 mRNA are measured relative to GAPDH. (B): Quantification of GFP-positive cells as a percent in time lapse imaging over 24 hours (h). (C): Representative images from (C) after 24 hours filming. (D): Representative stills from time lapse experiments (for full sequence refer Supporting Information Fig. S1). Arrows indicate the dying cell. wt: wild-type; p53+/−: deletion of one allele of p53; p53−/−: deletion of two alleles of p53; ko: Setd8-depleted cells; dko: double deletion of Setd8 and p53. Scale bars = 300 µm (C); 10 µm (D). Abbreviation: GFP, green fluorescent protein.

Setd8 causes cell death in vitro and in vivo [33,34]. However, we recently demonstrated that Setd8-induced apoptosis in the adult interfollicular epidermis can at least in part be rescued by simultaneous deletion of p53 [34]. Deletion of Setd8 during development is lethal and embryos with conditional deletion of Setd8 fail to develop the epidermis (Fig. 5A, 5B; upper and middle panels) [34]. Similar to our results in adult skin, we achieved a partial rescue of the embryonic skin defect by double deletion of Setd8 and p53 (Fig. 5A, 5B; lower panels). The p53-Setd8 double knockout mice (K14-GFP-dko) showed partial eyelid closure (Fig. 5A; asterisk) and developed skin, but the epidermis was fragile and peeled off (Fig. 5A; arrow). We next asked whether we could also rescue the contribution of Setd8-depleted bulge stem cells to wound healing in the absence of p53. However, the contribution of hair follicle bulge stem cells lacking Setd8 was minor and we very rarely observed GFP-positive (Setd8-deleted) cells in the epidermal wound of Setd8-p53 double knockout mice (K19-GFP-dko) (Fig. 5C; arrow). We obtained similar results when we cultured epidermal cells isolated from the single and double knockout mice (Fig. 6). We deleted Setd8 by applying Cre-recombinase to the culture medium and confirmed reduction of Setd8 expression as well as increase in GFP-reporter expression and absence of p53 (Fig. 6A). Time lapse live imaging of the epidermal cultures showed that Setd8-depleted cells survived longer when one (ko/p53+/−) or both alleles (dko) of p53 were ablated (Fig. 6B–6D; Supporting Information Fig. S1). Although p53-Setd8 double knockout cells (dko) even occasionally divided, they also eventually all died (Fig. 6D). Thus, rescue of Setd8 knockout by deletion of p53 may increase survival time but does not prevent cell death. These results underscore that cells are not able to survive in the absence of Setd8. We conclude that proliferating cells cannot survive in the absence of Setd8 in contrast to quiescent cells.

### Hair Follicle Stem Cells Require Setd8 to Survive Cell Division

**Figure 7:**
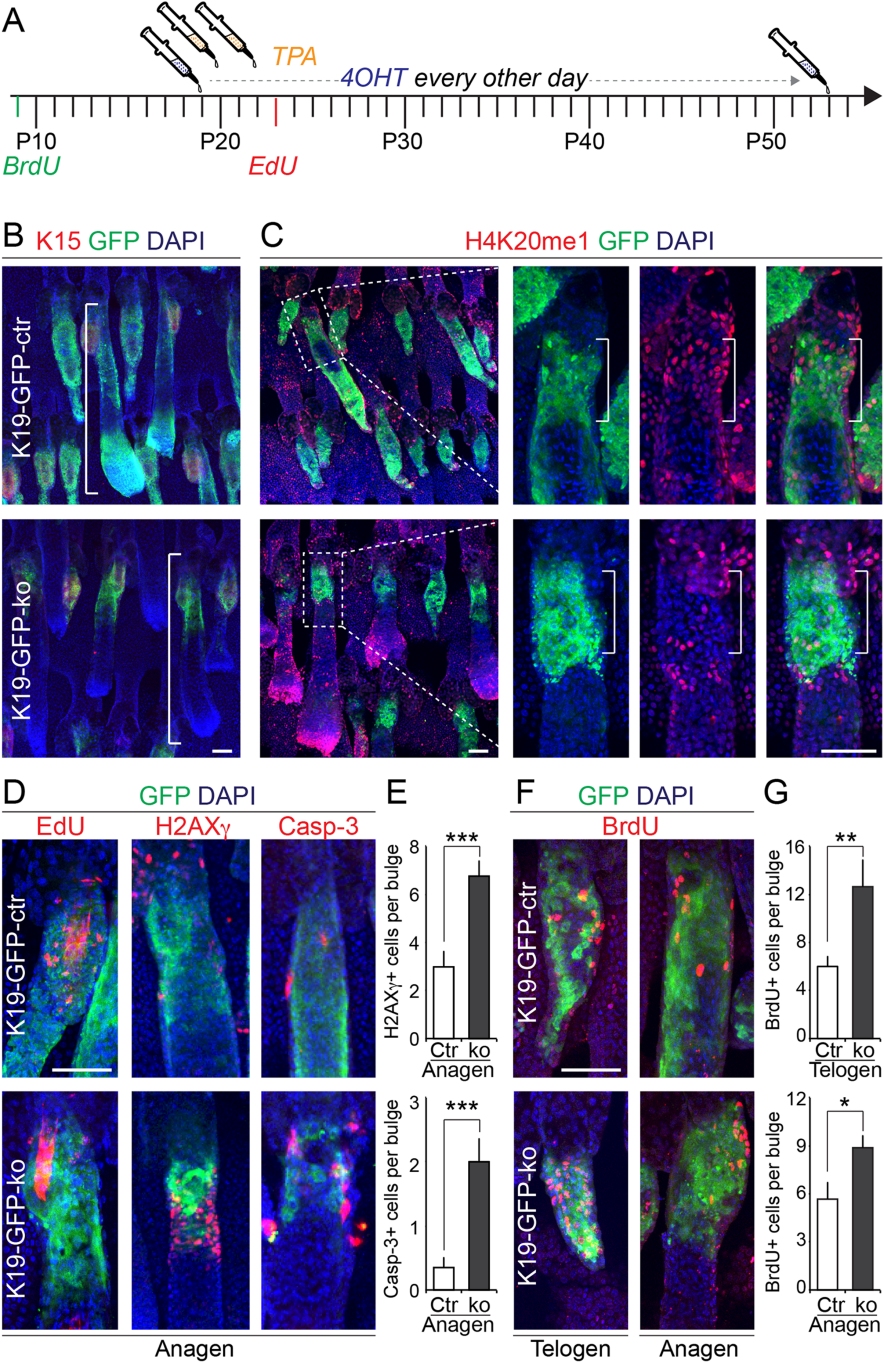
Hair follicle bulge cells require Setd8 to enter the cell cycle and contribute to hair regeneration. (A): Schematic diagram of experimental treatment with 4-OHT and TPA and the design to mark label-retaining cells (LRC) and proliferating cells in the hair follicle bulge using BrdU and EdU, respectively. (B): Coimmunofluorescence staining for keratin 15 (K15; red) and GFP (green) in skin of control (K19-GFP-ctr; upper panel) and Setd8-knockout (K19-GFP-ko; lower panel) mice. (C): Coimmunofluorescence staining for H4K20 monomethylation (H4K20me1) (red) and GFP (green) in skin of control (K19-GFP-ctr; upper panels) and Setd8-knockout (K19-GFP-ko; lower panels) mice. The three right hand panels show higher magnification of the boxed area in the left hand panels. (D): Coimmunofluorescence staining for EdU (left hand panels), H2AXγ (middle panels), and cleaved Caspase-3 (Casp-3; right hand panels) (red) with GFP (green) in control (K19-GFP-ctr; upper panels) and Setd8-knockout (K19-GFP-ko; lower panels) anagen hair follicles. (E): Quantification of H2AXγ and Casp-3-positive cells shown in (D). (F): BrdU (red) labeled LRCs in GFP-positive bulges of control (K19-GFP-ctr; upper panels) and Setd8-knockout (K19-GFP-ko; lower panels) mice showing telogen (left hand panels) or anagen (right hand panels) hair follicles. (G): Quantification of LCR cells in telogen (upper panel) and anagen (lower panel) hair follicles. Animals used for the experiment: 12 age-matched mice at the age of ∼3 weeks (9 K19-GFP-ctr; 3 K19-GFP-ko). Nuclei are counterstained with DAPI (blue) (B, C, D, F). Error bars represent SEM. *, *p* <. 05; **, *p* <. 005; ***, *p* <. 001. Scale bars = 50 µm (B–D, F). Abbreviations: 4-OHT, 4-hydroxy tamoxifen; GFP, green fluorescent protein.

To assess the effect of Setd8-deletion on the quiescent bulge stem cell population, we followed the fate of Setd8-depleted cells in vivo. We treated the tail skin with 4-OHT for 4 weeks and to recruit them into the cell cycle, we treated the skin with TPA twice (Fig. 7A). To mark the quiescent DNA label-retaining cells (LRCs) in the bulge, we injected BrdU at P10 and to label TPA-activated dividing stem cells we injected EdU once at P23 (Fig. 7A) [38]. As described earlier, the overall skin morphology was unaltered in both tail and back skin (Fig. 7B; Supporting Information Fig. S2A).

As expected, in control animals GFP-labeled bulge cells contributed to the whole hair follicle in tail skin (Fig. 7B; line; upper panel) and back skin (Supporting Information Fig. S2B). In contrast, GFP-labeled Setd8-negative cells failed to contribute to the growing hair and the population remained restricted to the upper part of the bulge (Fig. 7B; line; lower panel). To confirm that the distinct GFP-positive cell population was indeed Setd8-negative and remained quiescent in response to TPA treatment, we labeled the cells for monomethylated H4K20 (H4K20me1). H4K20me1 is absent in the quiescent bulge of telogen hair follicles [42]. While H4K20me1-positive cells were commonly found in GFP-labeled upper bulges of control hair follicles after TPA-induction (Fig. 7C; upper panels; lines), GFP-labeled H4K20me1-positive cells were rare in the absence of Setd8 (Fig. 7C; lower panels; lines).

The absence of EdU in Setd8-depleted bulges confirmed that quiescent stem cells failed to enter the cell cycle (Fig. 7D; left hand panels). Instead, we find a significant increase of apoptotic cells staining positive for gamma H2A.X (H2AXγ) or cleaved Caspase-3 (Casp-3) in the upper anagen bulge in the absence of Setd8 (Fig. 7D; middle and right hand panels; Fig. 7E). We also confirmed the increase in Caspase-3 and Tunel-positive cells in back skin hair follicles bulges of K19-GFP-ko mice compared to controls (K19-GFP-ctr) (Supporting Information Fig. S2C–S2E).

Notably, the lack of dividing bulge stem cells and the increase in cell death in the absence of Setd8 were not a stimulus for the quiescent bulge cells to enter the cell cycle because the number of LRCs was significantly increased in Setd8-depleted bulges in both telogen and anagen phases of the hair cycle (Fig. 7F, 7G). The higher number of LRCs and thus the lack of cell division in the bulge of K19-GFP-ko hair follicles (Supporting Information Fig. S2F, S2G) further indicated that hair follicle regeneration in the absence of Setd8 was not simply due to a few residual bulge cells that escaped ablation. We concluded that Setd8-depleted quiescent stem cells failed to enter the cell cycle and did not contribute to hair growth because of cell death, yet again the skin morphology was unaffected. Together, our data strongly indicate that other hair and/or epidermal populations can compensate for Setd8-induced cell death in bulge stem cells without disrupting skin morphology. Furthermore, our data also demonstrate that K19-expressing quiescent bulge stem cells are dispensable for skin homeostasis and regeneration after wounding.

## Discussion

The apparent lack of phenotype in response to Setd8-deletion in hair follicle bulge stem cells seems in stark contrast to the deleterious consequences of Setd8-ablation in epidermal progenitor cells [34]. Ectodermal progenitor cells fail to form a stratified epithelium in the absence of Setd8 and deletion of Setd8 in adult skin leads to the ablation of long-lived epidermal progenitors causing an irreversible loss of interfollicular epidermis and sebaceous glands [34]. The functional roles of Setd8 and H4K20me1 include many cellular pathways [33]. In all systems analyzed so far, loss of Setd8 causes cell cycle arrest and death because of its essential role in regulating chromosome stability during mitosis [27–32,34]. The robust induction of cell death in the absence of Setd8 is underscored by our observation that although deletion of p53 rescued from apoptosis in the short-term [34], cells with double deletion of Setd8 and p53 failed to survive in the longer term in vitro and in vivo.

However, even in Setd8-depleted epidermis loss of interfollicular epidermis and sebaceous glands was reversible and both structures can be regenerated from cells recruited from hair follicles [34]. These observations prompted us to use the essential role of Setd8 on cellular survival as a tool to specifically ablate quiescent bulge stem cells. Quiescent bulge stem cells represent the best candidate of a stem cell forming the apex of a potential hierarchical differentiation model.

While Setd8 is not required in quiescence, also K19-expressing bulge stem cells rely on Setd8 when they are recruited into the cell cycle and any contribution to hair and/or epidermal regeneration was blocked in the absence of Setd8. In addition to regulating cell cycle entry, Setd8 stimulates epithelial migration by turning off transforming growth factor (TGF) beta-induced Smad2 activation [43]. Therefore, deletion of Setd8 in the hair follicle bulge might also impair the migration of K19-positive stem cells out of the bulge at the beginning of anaphase or after wounding. However, none of the cellular defects observed in Setd8-depleted cells impaired hair follicle regeneration or wound healing.

While our data indicate that bulge cells are dispensable for hair follicle and epidermal regeneration, it does not imply that K19-expressing bulge cells are not important for both processes. Rather the opposite, to minimize the risk of organ failure it seems an important strategy to generate redundant backups in form of several cell populations with comparable plasticity.

Finally, we cannot exclude the possibility that induced cell death in the hair follicle bulge would affect skin homeostasis in the longer term. The ability of adult stem cells to reside in a quiescent state is considered to be crucial in preventing the exhaustion of stem cell populations [44–46]. Although we did not observe a reduction of quiescent bulge cells or major morphological differences in hair follicles or wound healing, we did measure a reduction of skin thickness and hair follicle length in the absence of bulge cell contribution. Notably, human skin decreases in thickness with age [47], and it is possible that a long-term inhibition of bulge stem cells would affect or accelerate the processes of aging. Bulge stem cell function decreases with age by displaying impaired self-renewal and a delayed response to regenerative stimuli [48,49].

## Conclusions

To address the question whether hair follicle stem cell populations are hierarchical organized, we blocked the contribution of K19-expressing quiescent bulge cells to hair and epidermal regeneration through conditional deletion of Setd8. Setd8 is required to maintain adult skin homeostasis and deletion of Setd8 epidermal progenitor cells causes cell death and sebaceous glands and interfollicular epidermis are lost [34]. Comparable to epidermal progenitors, also bulge cells cannot divide in the absence of Setd8 and undergo cell death. Accordingly, Setd8-depleted bulge cells fail to contribute to the regenerating hair follicle and epidermal wound healing. In contrast to deletion of Setd8 in epidermal progenitors, hair follicle morphology and wound healing process are unaffected in the absence of bulge cell contribution. Thus, bulge cells are dispensable for hair follicle and epidermal regeneration in the short term. In conclusion, K19-expressing bulge cells do not represent population at the apex of a stem cell hierarchy and are equipotent to other hair follicle stem cell populations.
