# Supplementary figures and images for: Genetically Induced Cell Death in Bulge Stem Cells Reveals Their Redundancy for Hair and Epidermal Regeneration
Source: Stem Cells. 2014 Dec 2;33(3):988–98. doi: 10.1002/stem.1910 (PMC4583782; doi:10.1002/stem.1910)

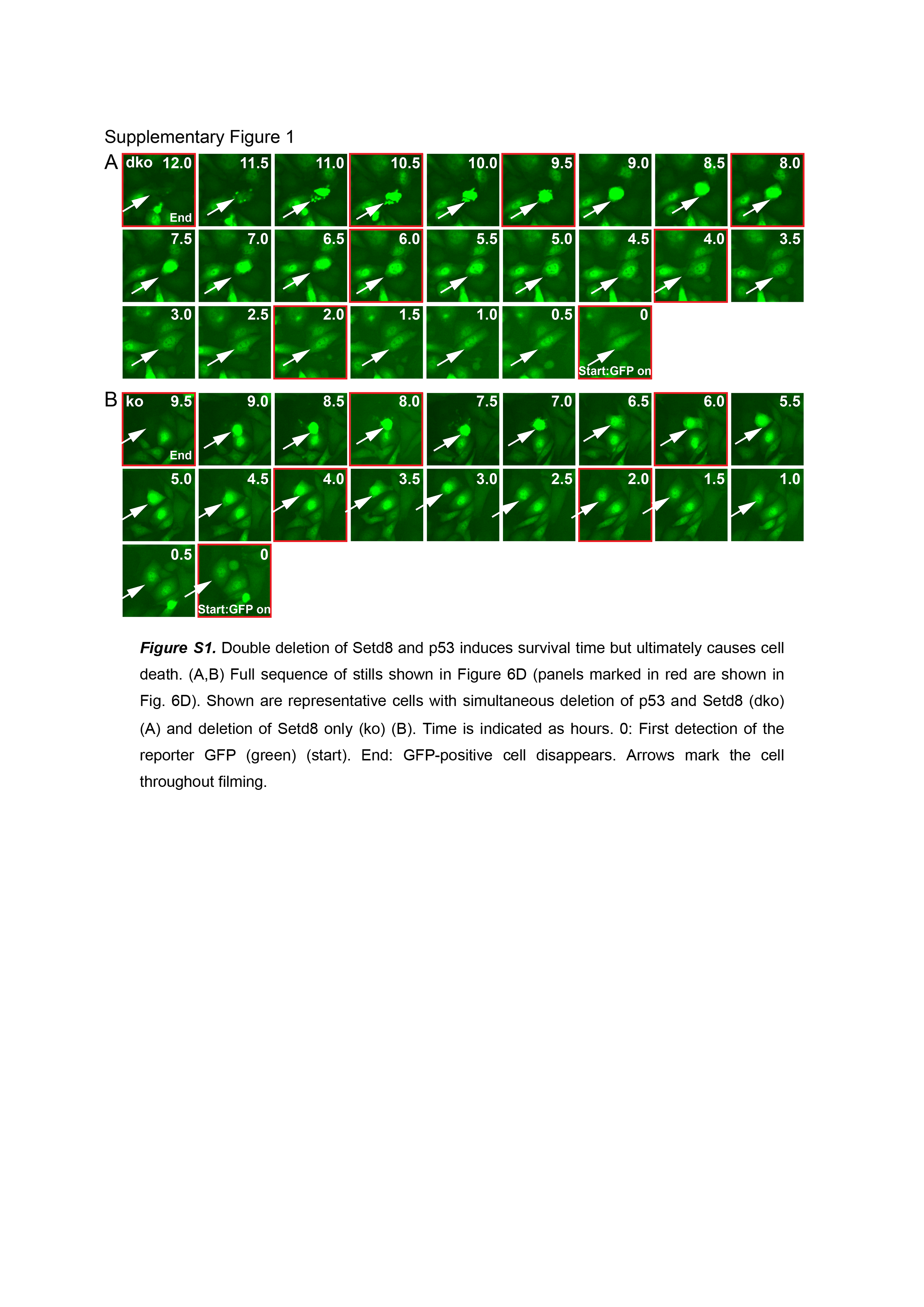

Supplement: Supplementary file 1 — Supplementary Information Figure 1 [file stem0033-0988-sd1.tif]

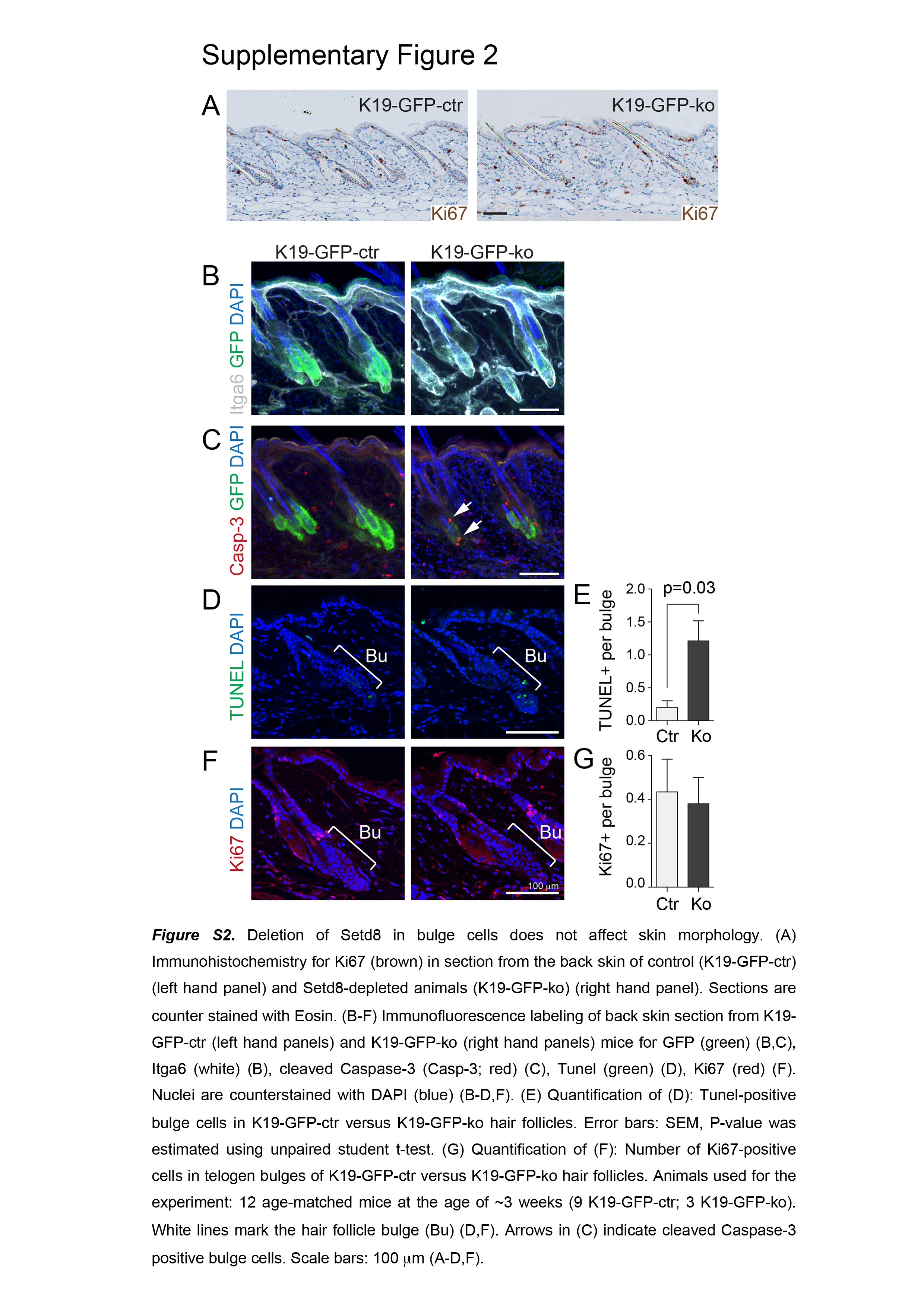

Supplement: Supplementary file 2 — Supplementary Information Figure 2 [file stem0033-0988-sd2.tif]
